# Tear Fluid Pharmacokinetics Following Oral Prednisone Administration in Dogs With and Without Conjunctivitis

**DOI:** 10.1089/jop.2019.0020

**Published:** 2019-07-24

**Authors:** Lionel Sebbag, Yuqi Yan, Joe S. Smith, Rachel A. Allbaugh, Larry W. Wulf, Jonathan P. Mochel

**Affiliations:** ^1^ Department of Veterinary Clinical Sciences, College of Veterinary Medicine, Iowa State University, Ames, Iowa.; ^2^ Department of Biomedical Sciences, SMART Pharmacology, College of Veterinary Medicine, Iowa State University, Ames, Iowa.; ^3^ Lloyd Veterinary Medical Center, College of Veterinary Medicine, Iowa State University, Ames, Iowa.; ^4^ Department of Veterinary Diagnostic and Production Animal Medicine, College of Veterinary Medicine, Iowa State University, Ames, Iowa.; ^5^ PhAST Laboratory, College of Veterinary Medicine, Iowa State University, Ames, Iowa.

**Keywords:** pharmacokinetics, prednisone, prednisolone, tear film, canine, blood–tear barrier

## Abstract

***Purpose:*** To describe the pharmacokinetics (PK) of prednisone and prednisolone in tear fluid of dogs receiving oral prednisone at anti-inflammatory to immunosuppressive doses and to assess the impact of induced conjunctivitis on lacrimal drug levels.

***Methods:*** Six healthy Beagle dogs were administered 4 courses of prednisone at 0.5, 1.0, 2.0, and 4.0 mg/kg given orally once a day for 5 days. At steady state, topical histamine was applied to induce mild (1 mg/mL) or severe (375 mg/mL) conjunctivitis in 1 eye of each dog and tear samples were collected from both eyes at selected times. Prednisone and prednisolone were quantified in tears by liquid chromatography-mass spectrometry.

***Results:*** Lacrimal prednisone and prednisolone concentrations ranged from 2 to 523 ng/mL and 5 to 191 ng/mL, respectively. Drug concentrations were overall greater in dogs receiving higher doses of prednisone, but were not correlated with tear flow rate. Eyes with conjunctivitis often had larger amounts of prednisone and prednisolone in tear fluid compared to control eyes (up to +64%), but differences were not statistically significant. Significantly greater, but clinically insignificant, levels of prednisolone were found in eyes with severe versus mild conjunctivitis for oral prednisone doses ≥1.0 mg/kg.

***Conclusions:*** Disruption of the blood–tear barrier with conjunctivitis did not significantly affect drug levels in tears. Based on drug PK in tears, oral prednisone is likely safe for the management of reflex uveitis and ocular surface diseases. However, further prospective trials using systemic corticotherapy in diseased animals are warranted to confirm findings from this preclinical study.

## Introduction

Prednisone is a corticosteroid with a wide range of pharmacological indications that is commonly used for the treatment of inflammatory and immune-mediated diseases in human and veterinary medicine. In ophthalmology, corticosteroids can alleviate ocular inflammation and help prevent devastating sequelae that could be painful or vision threatening.^1^ Of the various routes of administration, systemic therapy is generally recommended when the target tissue cannot be reached with topical ophthalmic corticosteroids (eg, eyelids, posterior segment, orbit), or as a complement to topical medications in cases of anterior uveitis.^2^

Systemically administered medications not only can readily distribute to the vascular tissues of the eye but can also affect the ocular surface if the drug reaches the tear compartment. For instance, oral doxycycline can be used as adjunctive therapy for keratomalacia in dogs and horses,^3,4^ while oral famciclovir is highly effective in managing herpetic keratoconjunctivitis in cats.^5,6^

For oral prednisone, detection of steroid levels in the tear film could support the use of systemic corticotherapy for adjunctive treatment of inflammatory diseases such as chronic superficial keratitis, immune-mediated keratitis, and keratoconjunctivitis sicca. Conversely, lacrimal levels of corticosteroids could inhibit corneal wound healing and potentiate infection.^7,8^ This therapeutic dilemma is exemplified in patients with ulcerative keratitis and concurrent reflex uveitis: systemic steroids are superior to nonsteroidal anti-inflammatory medications for controlling severe uveitis and preventing devastating sequelae,^9^ yet, ulceration could worsen and result in corneal perforation if wound healing is compromised and infection is potentiated.

The main goal of the study was to describe the pharmacokinetics (PK) of prednisone and its active metabolite prednisolone in tear fluid of dogs following oral administration at doses ranging from anti-inflammatory to immunosuppressive use (0.5–4 mg/kg/day). We hypothesized that prednisone and prednisolone would be quantifiable in canine tear fluid and concentrations would be greater with increasing oral dosing. In an effort to make the findings of this study more clinically relevant, a secondary objective was to determine the impact of conjunctivitis on drug concentrations in tears.

Indeed, conjunctivitis, a common bystander of most ocular diseases, increases conjunctival vascular permeability and therefore enhances vascular leakage of plasma compounds onto the ocular surface.^10,11^ We hypothesized that tear concentrations would be greater in eyes with conjunctivitis (ie, compromised blood–tear barrier) compared with healthy eyes.

## Methods

### Animals

Six Beagle dogs were included in the study. All were spayed females of 1.5–2 years old and weighing 7.5–10 kg. Before study inclusion, dogs were confirmed to be healthy based on physical and ophthalmic examination, complete blood count, serum chemistry, and urinalysis. The study was approved by the Institutional Animal Care and Use Committee of Iowa State University, and adhered to the Association for Research in Vision and Ophthalmology statement for the Use of Animals in Ophthalmic and Vision Research.

### Procedures

**FIG. 1.**
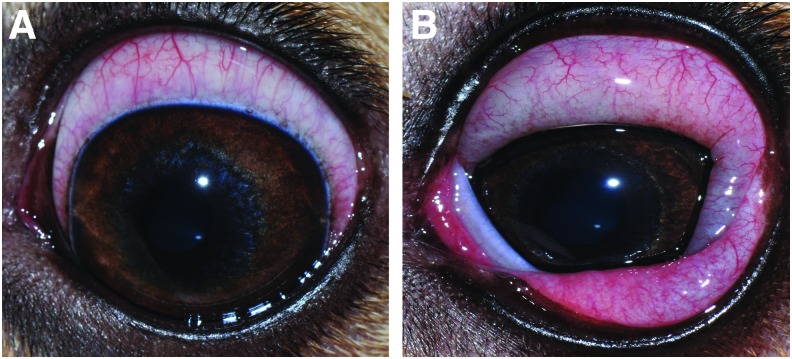
Topical histamine rapidly induced conjunctivitis (<1 min) that was either mild [**(A)**; 1.0 mg/mL solution] or severe [**(B)**; 375 mg/mL solution]. Color images are available online.

Over a period of 2 months, all dogs received 4 successive dosing regimens of oral prednisone (Cadista^TM^ predniSONE tablets; Jubilant Cadista Pharmaceuticals, Inc., Salisbury, MD), characterized by 5 days of drug administration interrupted by 9 days washout period, which included (1) 0.5 mg/kg once daily for 5 days; (2) 1.0 mg/kg once daily for 5 days; (3) 2.0 mg/kg once daily for 5 days; and (4) 4.0 mg/kg once daily for 5 days. The following procedures were performed on day 4 of each dosing regimen, a time chosen to ensure that steady state drug levels were reached^12^:

- Induction of conjunctivitis in 1 eye: Histamine ophthalmic solutions were formulated by mixing histamine powder (histamine dihydrochloride, FCC grade, Acros^®^ Organics, Geel, Belgium) with 1.4% polyvinyl alcohol lubricating eye drops (Artificial tears solution; Rugby, Rockville Center, NY) in a sterile manner under a laminar flow hood. Twenty minutes before prednisone administration, a single drop of histamine solution was applied to one randomly selected eye in each dog, while the other eye received artificial tears (Control). This ocular selection was kept constant throughout the study. Histamine rapidly induced conjunctivitis (<1 min) that was either mild (*n* = 3 dogs, 1.0 mg/mL histamine solution; Fig. 1A) or severe (*n* = 3 dogs, 375 mg/mL histamine solution; Fig. 1B), as previously described.^11^ Conjunctivitis was maintained throughout the 12 h collection period by repeating topical histamine administration every 1–4 h as needed to maintain effect. Topical 0.035% ketotifen fumarate (Zaditor^®^; Novartis Pharmaceuticals Corporation, East Hanover, NJ) was instilled onto each eye at the end of the day to control any residual conjunctival swelling.
- Tear collection: Tear fluid was sampled simultaneously in both eyes before prednisone administration (*t* = 0 min) and at 15, 30, 60, 90, 120, 240, 480, and 720 min following drug administration. The bent tip of a Schirmer tear strip (Eye Care Product Manufacturing, LLC, Tucson, AZ) was placed in the ventrolateral conjunctival fornix of each eye. While recording the test duration with a stopwatch, each Schirmer strip was removed and transferred into a 2-mL Eppendorf tube when the 20-mm mark of wetness was reached, as to standardize the volume of tears collected in each sample. The distal portion of each strip (25–35 mm marks, not wetted with tears) was spiked with 5 μL internal standard (prednisone-d7; Toronto Research Chemicals, North York, Canada) prepared as 10 ng/μL solution in 1:1 acetonitrile:water, and samples were stored at −80°C until analysis.
- Preparation of tear samples for analysis: The details of tear fluid extraction are described in the Appendix Table A1. In brief, both centrifugation and elution in solvent were used as complementary methods to extract the drug from Schirmer strips.^13^ Wetted strips containing tear fluid and internal standard were first centrifuged to retrieve the majority of absorbed tear fluid, followed by cutting and shredding the strips into small pieces and eluting them in methyl tert-butyl ether (MTBE). Of note, this particular solvent was chosen based on its superior ability to extract prednisone from Schirmer strips compared with methanol, acetonitrile, and water (small pilot study, data not shown).
- Liquid chromatography–mass spectrometry: Before study initiation, blank tears were collected from the same Beagle dogs using polyvinyl ophthalmic sponges as previously described.^14^ Eight standard curve solutions were prepared by spiking blank canine tears with stock solutions of prednisone/prednisolone (Cerrilliant, Round Rock, TX) to obtain the following concentrations: 1, 2, 5, 10, 20, 50, 100, and 200 ng/mL. Calibration curve samples were processed in a similar manner to biological tear samples, which involved wetting Schirmer strips with standard solutions until the 20-mm mark was reached, spiking prednisone-d7 internal standard onto the distal (dry) portion of the strips, centrifugation and elution in MTBE, and so on (see Appendix Table A1 for details). Concentrations of prednisolone and prednisone in canine tears were determined using high-pressure liquid chromatography (Agilent 1100 Pump, Column Compartment, and Autosampler, Santa Clara, CA) with ion trap mass spectrometry detection (LTQ; Thermo Scientific, San Jose, CA). The injection volume was set to 20 μL. The mobile phases consisted of A: 0.1% formic acid in water and B: 0.1% formic acid in acetonitrile at a flow rate of 0.25 mL/min. The mobile phase began at 20% B with a linear gradient to 95% B in 5.0 min, which was maintained for 2 min at 0.325 mL/min, followed by reequilibration to 20% B at 0.325 mL/min for 3.5 min. Separation was achieved with an ACE UltraCore C18 column, 100 mm × 2.1 mm, 2.5 μm particles (Mac-Mod Analytical, Chadds Cord, PA) maintained at 40°C. The chromatographic peaks for the internal standard, prednisone, and prednisolone (each eluted at 4.69 ± 0.05 min) were integrated using Xcalibur software (Thermo Scientific). Drug quantitation was based on linear regression analysis of calibration curves (weighted 1/X) using the analyte to internal standard area ratio. Calibration curves exhibited a correlation coefficient (*r*^2^) exceeding 0.995 across the concentration range. The limits of quantitation for prednisone and prednisolone were 2 ng/mL and 5 ng/mL, respectively, while the limits of detection for prednisone and prednisolone were 0.5 and 1 ng/mL, respectively.

### Data analysis

Noncompartmental analysis of prednisone and prednisolone PK was conducted with Phoenix software (WinNonlin, version 8.0; Pharsight Corporation, CA) to determine the maximum concentration (*C*_max_), time to maximum concentration (*T*_max_), and area under the curve from time zero to time of last measurable concentration (AUC_last_).

The Shapiro–Wilk test was used to assess data for normality. Non-normally distributed data were expressed as median and 95% central range (2.5–97.5th percentiles) and were analyzed with nonparametric statistics. Normally, distributed data are expressed as mean ± standard deviation (95% central range) and were analyzed with parametric statistics.

Associations between tear flow rate and lacrimal concentrations of prednisone or prednisolone were assessed with the Spearman's correlation test. The Student *t-*test was used to assess differences in AUC_last_ between eyes with mild or severe conjunctivitis. For each oral dose and for each PK parameter (AUC_last_, *C*_max_, *T*_max_), differences between control and conjunctivitis eyes (mild + severe) were assessed with the Student *t* test or Mann–Whitney test. For each PK parameter, differences among oral doses (0.5, 1.0, 2.0, and 4.0 mg/kg) were assessed with the one-way analysis of variance or Kruskal–Wallis test. Statistical analysis was performed using SigmaPlot 14.0 (Systat Software, Inc., San Jose, CA), and values *P* < 0.05 were considered statistically significant.

## Results

**FIG. 2.**
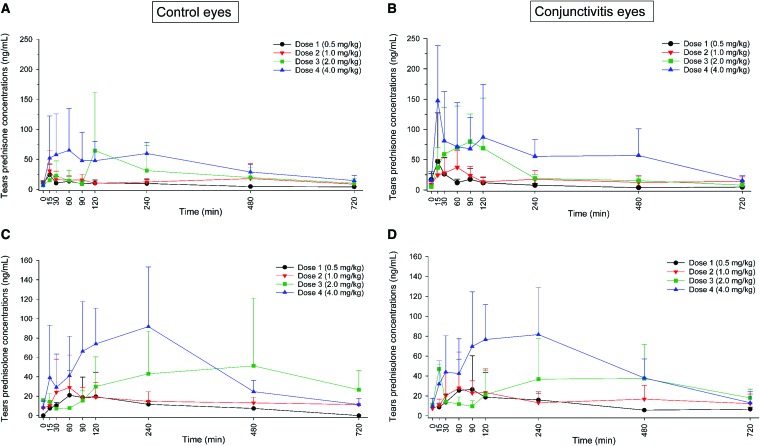
Tear film concentrations (mean + standard deviation) of prednisone **(A**, **B)** and prednisolone **(C**, **D)** in dogs receiving oral prednisone at 0.5 mg/kg once daily (*black line*, *circles*), 1.0 mg/kg once daily (*red lines*, *down triangles*), 2.0 mg/kg once daily (*green lines*, *squares*), and 4.0 mg/kg once daily (*blue lines*, *up triangles*). The concentrations are depicted for control eyes **(A**, **C)** and eyes with experimentally induced conjunctivitis **(B**, **D)**. Of note, the conjunctivitis group includes eyes with mild and severe conjunctivitis. Color images are available online.

**FIG. 3.**
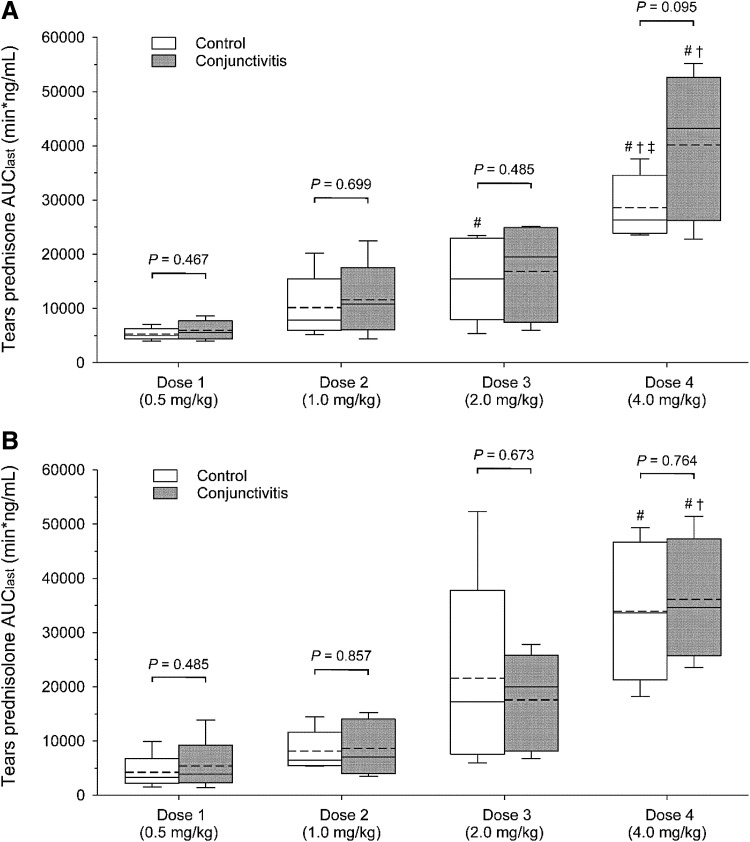
Box-and-whiskers plots depicting the area under the tear concentration–time curve from time zero to time of last measurable concentration (AUC_last_). Each plot depicts the mean (*dotted line*), median (*solid line*), 2.5th percentile (lower whisker), 25th percentile (lower limit of box), 75th percentile (upper limit of box), and 97.5th percentile (upper whisker). Data for prednisone **(A)** and prednisolone **(B)** are shown for all 4 oral doses of prednisone (0.5–4.0 mg/kg) in both control eyes (*white boxes*) and eyes with experimentally induced conjunctivitis (*dark gray*). Of note, the conjunctivitis group includes eyes with mild and severe conjunctivitis. Within the same drug dose, comparisons between control and conjunctivitis eyes (*t* test) are described with *P* values above the plots. Within the same ocular group (control or conjunctivitis), differences among doses (one-way analysis of variance) are depicted with *symbols* to demonstrate statistically greater AUC_last_ compared to dose 1 (#), dose 2 (†), and dose 3 (‡).

Following oral administration of prednisone, both prednisone and prednisolone were quantifiable in tear fluid, with concentrations ranging from 2 to 523 ng/mL and 5 to 191 ng/mL, respectively. Lacrimal concentrations were not correlated with tear flow rate for either prednisone (*P* ≥ 0.412) or prednisolone (*P* ≥ 0.388). However, higher doses of oral prednisone resulted in higher tear concentrations of both steroids, as depicted by the individual concentration–time curves in Fig. 2. In fact, the overall drug exposure in tears (depicted by AUC_last_) was statistically different among the 4 doses for both prednisone (*P* ≤ 0.002; Fig. 3A) and prednisolone (*P* ≤ 0.001; Fig. 3B). Similarly, statistical differences were detected among oral doses for prednisone *C*_max_ (*P* = 0.008) and for prednisolone *C*_max_ (*P* = 0.003) and *T*_max_ (*P* = 0.039; Table 1).

**Table 1.** Mean ± Standard Deviation of the Maximal Concentration (*C*_max_) and Time to Reach *C*_max_ (*T*_max_) for Prednisone and Prednisolone in Control and Conjunctivitis Eyes

	*Prednisone*		*Prednisolone*	
	*C_max_ (ng/mL)*	*T_max_ (min)*	*C_max_ (ng/mL)*	*T_max_ (min)*
Dose 1 (0.5 mg/kg)				
Control	23.2 ± 13.4	77.5 ± 89.7	19.2 ± 19.5	115.0 ± 99.3
Conjunctivitis	55.1 ± 68.1	87.5 ± 85.1	22.4 ± 26.7	130.0 ± 86.3
Dose 2 (1.0 mg/kg)				
Control	47.7 ± 26.4	140.0 ± 187.3	29.2 ± 24.9	215.0 ± 257.0
Conjunctivitis	48.1 ± 25.5	87.5 ± 85.1	37.1 ± 32.1	145.0 ± 166.9
Dose 3 (2.0 mg/kg)				
Control	91.7 ± 74.7	127.5 ± 97.6	75.3 ± 64.3	420.0 ± 211.3^#^
Conjunctivitis	137.2 ± 115.5	65.0 ± 22.6	62.7 ± 31.3	320.0 ± 196.0
Dose 4 (4.0 mg/kg)				
Control	116 ± 67.2^#^	127.5 ± 93.8	123.6 ± 47.8^#,†^	160.0 ± 92.3
Conjunctivitis	204.3 ± 173.5	120.0 ± 180.7	120.0 ± 38.1^#,†,‡^	160.0 ± 92.3

Within the same drug dose, comparisons between control and conjunctivitis eyes did not reveal any statistical differences (*P* > 0.05). Within the same ocular group (control or conjunctivitis), differences among doses are depicted with symbols to demonstrate statistically greater values compared to dose 1 (#), dose 2 (†), and dose 3 (‡).

**FIG. 4.**
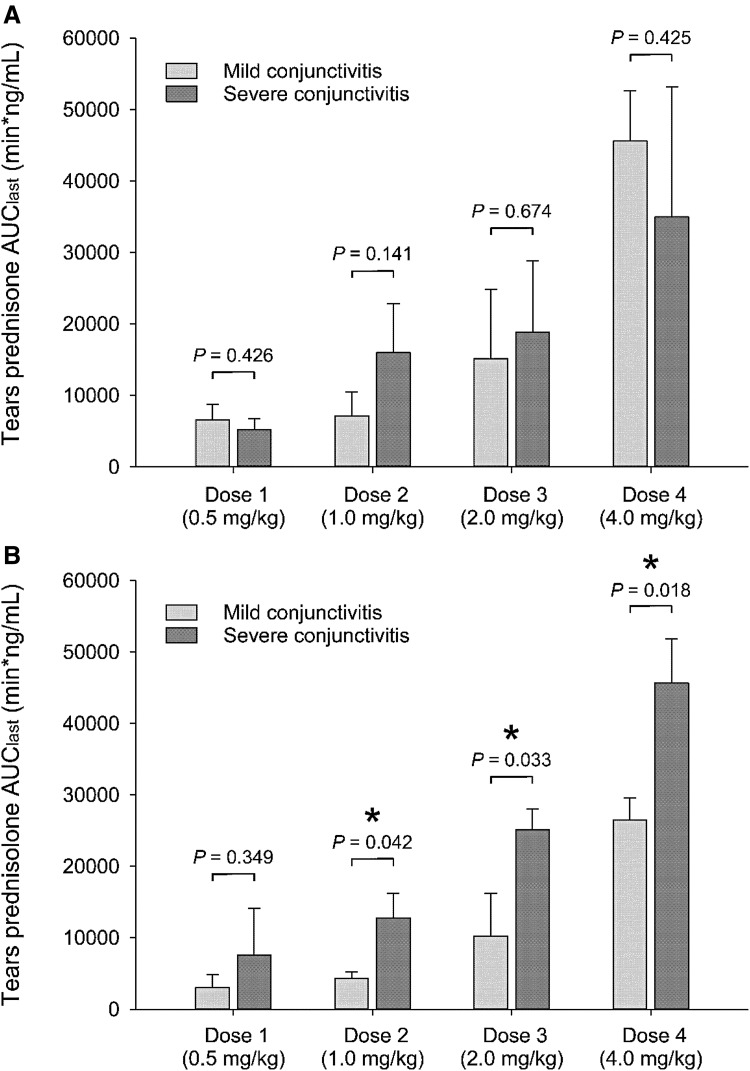
Bar charts depicting mean + standard deviation of area under the tear concentration-time curve from time zero to time of last measurable concentration (AUC_last_). Data for tear concentrations of prednisone **(A)** and prednisolone **(B)** are shown for all 4 oral doses of prednisone (0.5–4.0 mg/kg) in eyes with experimentally induced mild conjunctivitis (*light gray*) or severe conjunctivitis (*dark gray*). Within the same dose, statistical comparisons between mild and severe conjunctivitis (*t* test) are described with *P* values above the plots, and statistical significance (*P* < 0.05) is depicted with an *asterisk* (*).

In general, eyes with conjunctivitis showed a trend for larger concentrations of prednisone and prednisolone in tear fluid compared with control eyes (Figs. 2 and 3), with differences in average concentrations ranging from +5% to +64%. However, differences in AUC_last_ between control and conjunctivitis eyes were not statistically significant for either prednisone (*P* ≥ 0.095; Fig. 3A) or prednisolone (*P* ≥ 0.485; Fig. 3B). The severity of conjunctivitis did have an impact on lacrimal concentrations, as significantly greater levels of prednisolone were found in eyes with severe versus mild conjunctivitis for oral doses 1–4 mg/kg/day (*P* ≤ 0.042; Fig. 4), although these changes were not considered to be large enough to be clinically relevant.

## Discussion

The present study describes, for the first time, the tear film PK of prednisone and prednisolone in veterinary medicine. Tear film concentrations of prednisone and prednisolone varied from 2 to 523 ng/mL and 5 to 191 ng/mL, respectively, with higher doses of oral prednisone leading to higher lacrimal levels of both steroids. The question remains whether concentrations of the active metabolite (prednisolone) are relevant in clinical patients, that is potentially beneficial or detrimental to managing ocular surface diseases.

Overall, tear film prednisolone levels were ≥10^−9^ M (ie, 0.4 ng/mL) in all dogs throughout the 12-h sampling time, a concentration shown to decrease the expression of deleterious cytokines (tumor necrosis factor-α, interleukin-6) and matrix metalloproteinases in a rat model of keratitis.^15^ Therefore, oral prednisone might have therapeutic benefits in managing corneal inflammation, assuming similar exposure-response between species.

In fact, although the use of corticosteroid in infectious keratitis remains controversial,^16^ some authors believe that a judicious use of corticosteroids (combined with the appropriate antimicrobial) could improve the outcome of keratitis as it reduces damage caused by the host's inflammatory response, decreases corneal scarring, and inhibits neovascularization.^17,18^ From a safety viewpoint, the use of corticosteroids is known to potentially delay corneal wound healing and exacerbate signs of ocular infection. *In vitro*, inhibition of corneal wound healing in dogs is only reported for prednisolone concentrations that are much higher (≥620 μg/mL)^19^ than the ones reported herein. *In vivo*, topical corticosteroid use can be detrimental in patients with ulcerative keratitis^2^ although tear film concentrations following topical 1% prednisolone acetate are unknown to date in any species. Data extrapolation from PK of 0.3% ciprofloxacin in dogs^20^ show that (1) topical 1% prednisolone acetate could reach concentrations as high as 909 μg/mL, which is 1,000- to 10,000-fold greater than drug levels noted in the present study; and (2) topical 1% prednisolone acetate (applied every 6 h) could result in drug exposure over 12 h that is 14,000–27,000-fold and 3,300–5,300-fold greater than oral prednisone given at anti-inflammatory dose (0.5–1 mg/kg/day) or immunosuppressive dose (2–4 mg/kg/day), respectively.

Of note, drug exposure over time is more relevant than single lacrimal concentrations (eg, *C*_max_) given differences in pharmacological disposition between topical and oral routes. While the ocular bioavailability of topical administration is <10%–20% given efficient washout by tears,^21^ oral administration could be considered as a form of sustained-release at the ocular surface through lacrimal gland diffusion and conjunctival leakage.

As for the negative impact on the immune system, there is no consensus on what concentration is considered harmful. In one study, prednisolone levels as low as 0.005 μg/mL were shown to reduce the phagocytosis function of human leucocytes,^22^ while prednisolone concentrations as high as 4.32 μg/mL did not impact leucocyte phagocytosis or bactericidal activity in another study.^23^

Conjunctivitis is a common disorder in dogs that develops concurrently to most ocular diseases, whether affecting the adnexa (eg, blepharitis), ocular surface (eg, corneal ulcer), or intraocular tissues (eg, uveitis). With conjunctivitis, plasma constituents tend to “leak” into the tear compartment as the permeability of conjunctival vessels is typically increased.^10,11^ This breakdown of the blood–tear barrier explains the large quantities of albumin in tears of diseased eyes, regardless of the underlying etiology of conjunctivitis (eg, dry eye, corneal ulcer, allergies).^24,25^ Thus, to make the present PK findings more clinically relevant, conjunctivitis was experimentally induced in selected canine eyes using a recently described model.^11^

Lacrimal levels of prednisone and prednisolone were overall higher in conjunctivitis versus control eyes, with greater disease severity leading to generally greater drug levels in tears, especially for prednisolone. However, differences between control versus conjunctivitis eyes were not statistically significant, and were fairly minimal (up to 64% increase) when compared to plasma albumin (up to 12,000%).^11^ Unlike albumin, a very large molecule (66,500 Da) that does not permeate through intact conjunctival tissue,^24^ we suspect that smaller molecules like prednisone (358 Da) and prednisolone (360 Da) readily cross the blood–tear barrier under normal conditions, and are therefore not significantly impacted by conjunctival inflammation.

However, we cannot exclude that larger amounts of corticosteroids actually reach the lacrimal fluid in eyes with conjunctivitis; although the concurrent leakage of albumin binding to free prednisolone would probably reduce its bioavailability at the ocular surface.^26^ Further, physicochemical properties other than molecular weight may explain differences in lacrimal distribution between prednisone, prednisolone, and other drugs reported in the veterinary literature^3,5,13^—namely protein binding, lipophilicity, and degree of ionization.^27^ In this study, the competitive nature of plasma protein binding between prednisone and prednisolone could justify the slightly higher lacrimal concentrations of prednisone in canine tears.^28^

The present study has a few limitations. First, the sample size of our experiment was relatively small, and only females from a single dog breed were evaluated. Tear film PK could theoretically differ in male versus female dogs,^29^ or breeds other than Beagle, especially in brachycephalic dogs in whom the lacrimal lipid layer is thin and corneal exposure is large.^30^ Yet, previous studies did not find significant differences between mesocephalic and brachycephalic dogs with regard to tear film dynamics (tear volume, tear turnover rate)^31^ or tear film drug concentrations.^20^

Second, it is possible that we did not find statistical differences in tear film concentrations between healthy versus conjunctivitis eyes because of the conjunctivitis model itself. Experimental induction of conjunctivitis, although rapid and noninvasive,^11^ could have falsely lowered lacrimal concentrations by causing reflex tearing and accelerated tear turnover. We minimized this risk by inducing conjunctivitis ≥20 min before drug administration and sample collection, but a small degree of ocular irritation could have lingered. Regardless, increased tearing should not have affected drug levels in a notable manner, as we did not find a significant correlation between tear flow rate and prednisone/prednisolone levels in tears.

Finally, the present study used Schirmer strips to collect tears in dogs, and although this method yielded sufficient tear fluid for analysis, the method has several disadvantages that could partly explain the large variability in tear concentrations noted among subjects. Not only do Schirmer strips absorb tear fluid but they also retain a certain amount of tear components (adsorption), the degree of which can vary depending on the concentration.^13^

In this study, we minimized the impact of adsorption by incorporating 2 important steps in our sample preparation: (1) the internal standard was spiked onto the distal end of Schirmer strips before tear extraction, and (2) standard curves were processed in a similar manner to biological samples. We also maximized the amount of drug extracted from Schirmer strips by combining centrifugation with solvent elution.^13^ Such combination may improve the assay sensitivity (ie, able to detect lower concentrations), but the process is very labor intensive and may not be necessary for all drugs. Future studies should consider a pilot experiment to assess the extraction efficacy of the combination method versus centrifugation or solvent elution alone.

In conclusion, our data indicate that oral prednisone might be safe and beneficial as adjunctive therapy for reflex uveitis, ulcerative keratitis, or other ocular surface disease in dogs. However, these preliminary pharmacokinetic findings need to be complemented with prospective controlled studies using systemic corticotherapy in diseased animals. Similarly, future anatomical and physiological studies are needed to better understand the role of conjunctivitis in diffusion of systemically administered drugs into the tear film.

## Appendix

**Appendix Table A1.** Preparation of Schirmer Strips for Liquid Chromatography-Mass Spectrometry Analysis

1	Transfer the Schirmer strip (wetted until 20-mm mark) to a 2-mL tube^a^ using single-use tweezers^b^
2	Spike 5 μL of 10 ng/μL prednisone internal standard^c^ (10 ng/μL prepared in 1:1 acetonitrile:water) on the dry portion of the strip (ie, 25–35 mm strip end)
3	Place the Schirmer strip into a 0.2-mL tube^d^ that was prepunctured at its bottom with a 18-gauge needle
4	Secure the 0.2-mL tube into a 2-mL tube using adhesive tape
5	Centrifuge^e^ the combination for 2 min at 3,884 *g* to extract tear fluid out of the Schirmer strip into the 2-mL tube
6	Transfer the centrifuged strip to another 2-mL tube and cut it into multiple <5 mm pieces
7	Add 600 μL of methyl tert-butyl ether (MTBE) into the tube containing the cut Schirmer strip
8	Grind for 1 min with a handheld pestle^f^
9	Store in +4°C fridge for 2 h
10	Ultrasonic agitation^g^ for 30 min
11	Centrifuge^h^ for 1 min at 3,824 *g*
12	Transfer the fluid to a new 2-mL tube, leaving the cut/shredded Schirmer strip behind
13	Dry solvent with nitrogen^i^ for 6–8 min at 5–8 psi
14	Add 75 μL of 25% acetonitrile
15	Vortex^j^ for 1 min
16	Centrifuge^h^ for 1 min at 3,824 *g*
17	Transfer the fluid to the 2-mL tube containing the centrifuged tear sample (step #5)
18	Vortex^j^ for 30 s
19	Centrifuge^h^ for 30 s at 3,824 *g*
20	Transfer into LC-MS vial^k^ containing a glass insert and a snap cap
21	Centrifuge^l^ at 1,074 *g* for 10 min
22	Transfer the sample from the glass insert to a 2-mL tube
23	Add 320 μL of iced cold 100% acetonitrile
24	Centrifuge^h^ for 10 min at 10,621 *g*
25	Transfer to a new 2-mL tube, leaving the precipitated proteins behind
26	Dry solvent with nitrogen^i^ for 10–15 min
27	Add 100 μL of 25% acetonitrile
28	Vortex^j^ for 5 s
29	Transfer the sample to a new glass insert
30	Centrifuge^l^ at 998 *g* for 15 min

Steps 23–30 were added after the initial samples were deemed inappropriate for LC-MS analysis. The proteins contained in the centrifuged tear sample were clogging the LC columns, so further precipitation in acetonitrile was required.

^a^ Two milliliter cryogenic vial; Fisherbrand, Fisher Scientific, Pittsburgh, PA.

^b^ Evident^®^ disposable plastic tweezer; Evident, LLC, Union Hall, VA.

^c^ Prednisone-d7; Toronto Research Chemicals, Inc., North York, Canada

^d^ Eppendorf^®^ safe-lock micro test tubes; Eppendorf North America, Inc., Westbury, NY.

^e^ VWR^®^ mini centrifuge; VWR International, Mississauga, Ontario, Canada.

^f^ Argos^®^ pestle mixer and pestle; Argos Technologies, Inc., Elgin, IL.

^g^ Bransonic^®^ ultrasonic cleaner; Branson Ultrasonics Corporation, Danbury, CT.

^h^ Eppendorf^®^ Centrifuge 5417C; Eppendorf North America, Inc., Hamburg, Germany.

^i^ Biotage^®^ nitrogen evaporator; Biotage, LLC, Charlotte, NC.

^j^ LP vortex mixer; Thermo Scientific, Inc., Waltham, MA.

^k^ Xpertek^®^ 2-mL 12 mm × 32 mm snap seal vial; P.J. Cobert Associates, Inc., St. Louis, MI.

^l^ Sorvall ST40 centrifuge, Thermo Scientific, Inc., Waltham, MA.

LC-MS, liquid chromatography-mass spectrometry.
